# Lysosome targeted therapies in hematological malignancies

**DOI:** 10.3389/fonc.2025.1549792

**Published:** 2025-02-24

**Authors:** Madhumita S. Manivannan, Anthea Peters, Spencer B. Gibson

**Affiliations:** Department of Oncology, Faculty of Medicine and Dentistry, University of Alberta, Edmonton, AB, Canada

**Keywords:** lysosomes, autophagy, hematological malignancies, lysosomal dysfunction, chemotherapy resistance, targeted treatment, drug resistance, leukemia

## Abstract

Lysosomes are dynamic organelles integral to cellular homeostasis, secretory pathways, immune responses, and cell death regulation. In cancers, lysosomes become dysregulated to sustain proliferative signaling, metabolism, and invasion. In hematological malignancies such as acute myeloid leukemia (AML), acute lymphoblastic leukemia (ALL), chronic myeloid leukemia (CML), and chronic lymphocytic leukemia (CLL), leukemia cells demonstrate lysosome dysregulation with increased lysosomal activity, mTORC1 signaling, catabolic reactions, and autophagy. This supports the survival, metabolism, and proliferation of the leukemia cells. Lysosomes also play a critical role in treatment resistance by promoting cell survival and sequestration of drugs. This has led to the development of lysosome-targeted therapies such as cationic amphiphilic drugs (CAD), ATPase inhibitors or autophagy inhibitors to treat hematological malignancies. Lysosome-targeted treatments have shown effectiveness at inducing cell death by inhibiting cell survival mechanisms and inducing apoptosis. In addition, the combination of lysosome-targeted therapies with standard treatments gives synergistic apoptotic responses in leukemia cells. In this review, we will describe the lysosomal functions, their dysregulation in hematological malignancies and the development of lysosomal targeted therapies for leukemia treatment. By understanding lysosome dysregulation and developing lysosome-targeted agents, innovative treatment strategies could be effective in overcoming drug resistance in hematological malignancies.

## 1 Background

Lysosomes, membrane-bound organelles rich in hydrolytic enzymes, are essential for degrading and recycling cellular waste. Discovered by Christian de Duve in the 1950s, lysosomes were initially characterized as the cell’s digestive system (1). However, their roles extend far beyond degradation, encompassing processes such as autophagy, antigen presentation, secretion, and regulation of cell death (2–4). These multifaceted functions are crucial for maintaining cellular homeostasis and responding to various stressors (5).

Lysosomes have garnered significant attention in hematological malignancies such as leukemia due to their involvement in cancer cell metabolism, survival, and resistance to therapy (6). Leukemias are cancers of the blood, bone marrow, and lymphatic systems characterized by the uncontrolled proliferation of malignant cells. Traditional treatments, including chemotherapy and radiation, often face challenges such as drug resistance and adverse side effects. This has driven the search for novel therapeutic targets to improve treatment efficacy and patient outcomes.

Lysosomes play a pivotal role in the pathology of these malignancies through their involvement in autophagy, a cellular process that cancer cells exploit for survival under stress conditions, including nutrient deprivation and chemotherapy (7). Dysregulation of lysosomal pathways can lead to altered autophagy, contributing to cancer progression and resistance to treatment (8). Additionally, lysosomal enzymes and their role in apoptosis regulation are critical in determining the sensitivity of cancer cells to therapy-induced cell death.

Targeting lysosomal functions and pathways offers a promising therapeutic strategy in hematological malignancies. Inhibitors of autophagy, lysosomal membrane permeabilization, and enzyme activity are being explored to enhance the efficacy of existing treatments and overcome drug resistance (9). By disrupting the lysosomal machinery that cancer cells rely on, these therapies selectively induce cell death in malignant cells while sparing normal cells.

Understanding the role of lysosomes in hematological malignancies’ pathogenesis and therapy resistance is crucial for developing targeted treatments. Ongoing research is focused on elucidating the molecular mechanisms governing lysosomal functions and identifying potential therapeutic targets within these pathways. This approach holds promise for improving the prognosis and quality of life for patients with hematological malignancies, highlighting the lysosome’s potential as a key player in cancer therapy. This review will summarize the current understanding of lysosomal functions in general and in hematological malignancies, specifically leukemia, and explore the potential of targeting lysosomal pathways as a novel therapeutic strategy for these cancers.

## 2 Lysosome functions

### 2.1 Lysosomal characteristics

Lysosomes are membrane-bound organelles characterized by their acidic interior, which is maintained by the vacuolar H+-ATPase (V-ATPase) that actively pumps protons into the lysosome (10). This acidic environment is essential for the optimal activity of lysosomal enzymes and for the degradation of macromolecules. The lysosomal membrane is composed of a lipid bilayer enriched with cholesterol and specific lysosomal membrane proteins such as lysosome-associated membrane proteins (LAMP-1 and LAMP-2). These proteins play crucial roles in maintaining lysosomal integrity, facilitating the transport of materials, and protecting the lysosome from autolysis (11). The membrane also contains transporters and ion channels, including those for calcium and protons, which regulate lysosomal function and signalling.

Lysosomes house over 60 hydrolases, including proteases, lipases, nucleases, and glycosidases (2). Key proteolytic enzymes include cathepsins (e.g., cathepsin B, D, and L), which degrade proteins into amino acids. These enzymes are synthesized as inactive precursors and activated in the acidic lysosomal environment. Their coordinated activity enables the breakdown of diverse substrates, from damaged organelles to extracellular material.

**Figure 1:**
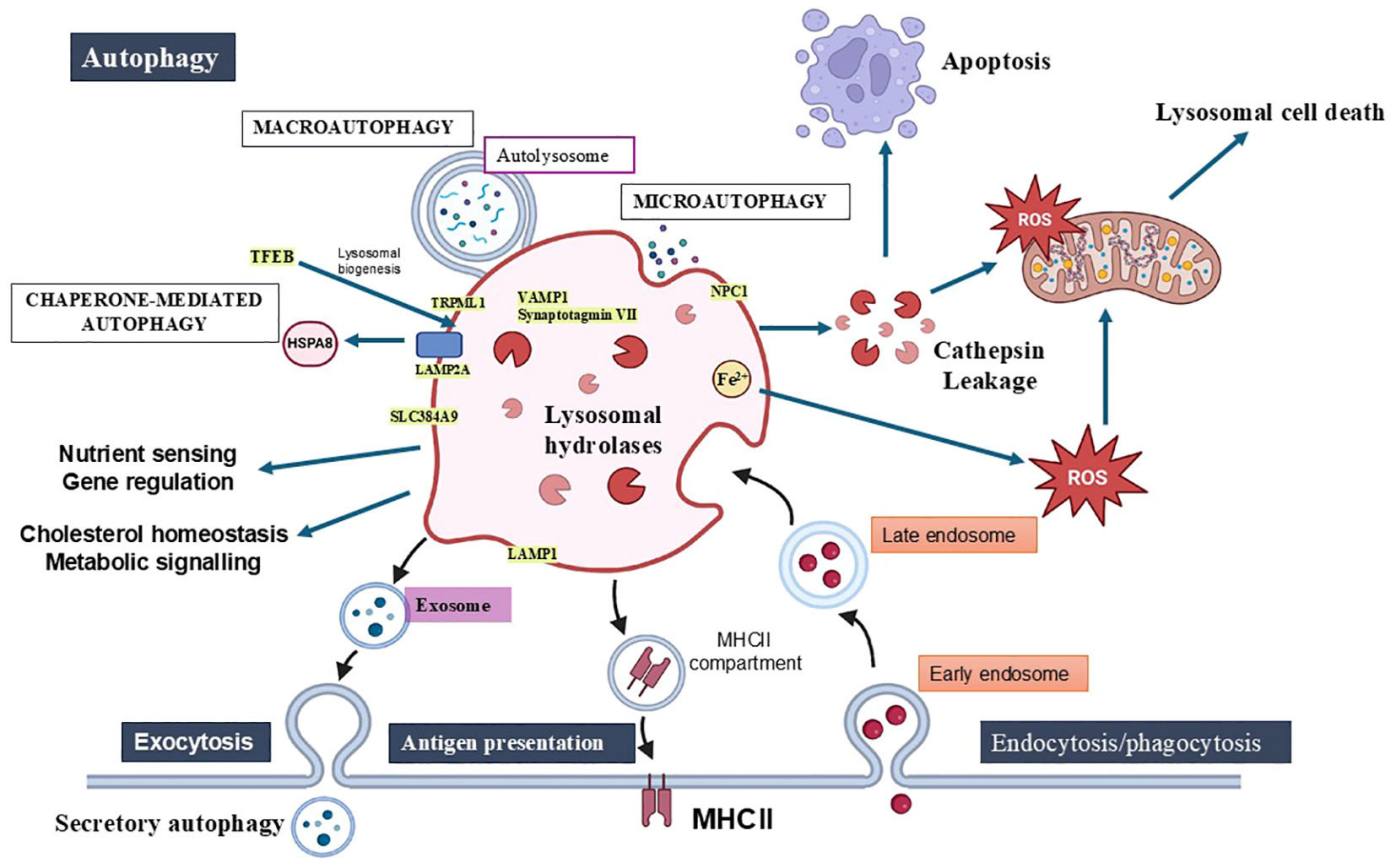
Lysosomal functions in cellular homeostasis, autophagy, immunity, and cell death. Lysosomes play key roles in maintaining homeostasis by degrading and recycling macromolecules, supporting nutrient sensing, cholesterol regulation, metabolic signalling, and energy homeostasis. The three types of autophagy—macroautophagy, microautophagy, and chaperone-mediated autophagy—are essential for removing damaged organelles and intracellular pathogens. Lysosomes also contribute to immune functions through phagocytosis, antigen processing via MHC-II and lysosomal exocytosis. Lysosomal membrane permeabilization (LMP) is a pivotal event that triggers various forms of cell death, including apoptosis, necrosis, pyroptosis, and ferroptosis, through the release of hydrolases, cathepsins, and reactive oxygen species (ROS). This figure highlights lysosomes’ dual role in promoting both cellular survival through autophagy and inducing cell death when lysosomal integrity is disrupted. Key components of the lysosomal membrane include integral proteins (LAMP1, LAMP2A), ion channels (TRPMLs), trafficking and fusion proteins (Synaptotagmin VII, VAMP7), lipid and amino acid transporters (NPC1, SLC38A9).

Lysosomes are central to cellular signalling pathways, including those regulating autophagy, metabolism, and immune responses. The mechanistic target of rapamycin complex 1 (mTORC1) is a critical lysosome-associated signalling hub. mTORC1 senses nutrient availability and regulates cell growth and autophagy (12). Additionally, lysosomes interact with transcription factors such as TFEB (transcription factor EB), which governs lysosomal biogenesis and autophagy by responding to cellular stress and nutrient signals (13) (**Figure 1**).

Calcium ions play a vital role in lysosomal dynamics and signalling. Lysosomes act as calcium stores, releasing calcium through channels like TRPML1 (transient receptor potential mucolipin 1) in response to stimuli. This calcium release is critical for processes such as vesicle trafficking, fusion with other organelles, and activation of signalling cascades (14). Dysregulation of lysosomal calcium signalling is implicated in various diseases, including neurodegenerative disorders and cancer (15).

By integrating these characteristics, lysosomes emerge as multifunctional organelles pivotal to cellular health and disease, making them promising targets for therapeutic intervention in conditions like leukemia and lymphoma.

### 2.2 Cellular homeostasis

Cellular homeostasis involves the synthesis, folding, trafficking, and degradation of proteins, lipids, nucleic acids, and sugars crucial for cellular function and survival (16). Lysosomes play a critical role in degrading and recycling these cellular macromolecules or redundant/damaged organelles (17). For example, lysosomes provide amino acids and glucose, which promote the translocation of mTORC1 to the lysosomal surface, mediating cellular and organismal growth (18, 19). In addition, lysosomes also digest extracellular material supplied by endocytosis or phagocytosis. In leukemia and other cancers, lysosomal dysfunction can disrupt cellular homeostasis, impairing the degradation and recycling of essential macromolecules and damaging organelles (20). This dysfunction can lead to metabolic adaptations that support cancer cell growth, survival, and resistance to treatments, as lysosomes provide key nutrients like amino acids and glucose, which fuel processes such as mTORC1 activation (21).

#### 2.2.1 Role of lysosome in digestion

Lysosomes play a direct role in cellular homeostasis through the degradation of macromolecules. This is accomplished through lysosomes containing many acid hydrolases such as proteases, nucleases, glycosidases, lipases, phospholipases, phosphatases, peptidases and sulfatases responsible for this degradation. Lysosomes have membrane proton pump proteins of the H+-ATPase family that ensure an acidic pH in the lysosome (10). These proteins are highly glycosylated protecting them from lysosome protease degradation. This provides an acidic pH in lysosomes to maintain acidic hydrolase enzymatic activity. The lysosome membrane also contains transport proteins that transfer the degraded products to the cytosol for secretion or reuse by the cell.

Lysosomal positioning within the cell is also important for coordinating catabolic and anabolic processes in response to nutrient availability. Under nutrient-rich conditions, lysosomes are found at the cell periphery, associated with activated mTORC1. In contrast, during starvation, lysosomes cluster around the perinuclear region, facilitating the fusion of autophagosomes with lysosomes and the subsequent release of nutrients (22). This strategic positioning allows lysosomes to effectively manage nutrient sensing and energy homeostasis.

Lysosomal hydrolases play a direct role in the mobilization of energy stores, digesting and mobilizing nutrients under growth-promoting conditions. However, when undigested lipids or glycogen accumulate inside lysosomes, they become toxic, leading to pathological states ranging from mild disease to death (23, 24). By degrading macromolecules and mobilizing nutrients like amino acids and glucose, lysosomes support cellular energy homeostasis, fueling cancer cell proliferation and survival (25). In nutrient-rich conditions, lysosomes’ interaction with activated mTORC1 promotes cell growth and metabolic reprogramming, a common feature in cancer cells. Conversely, during nutrient deprivation or stress, lysosomes facilitate autophagy, allowing cancer cells to adapt by recycling cellular components for energy (26). However, dysfunction in lysosomal hydrolases, transport proteins, or membrane acidification can lead to the accumulation of undigested materials, contributing to cellular toxicity and promoting tumour progression (27). Furthermore, altered lysosomal positioning and dysfunction in nutrient sensing can exacerbate metabolic imbalances in cancer cells, enhancing their resistance to therapies such as chemotherapy and radiation (8). The central role of lysosomes in maintaining nutrient homeostasis and regulating cellular metabolism underscores their potential as therapeutic targets in cancer.

#### 2.2.2 Role of lysosomes in autophagy

Autophagy is a process that maintains cellular homeostasis. There are three main types of autophagy: macroautophagy, microautophagy, and chaperone-dependent autophagy. Microautophagy is a non-selective process in that fragments of the cytoplasm are absorbed by lysosomes by invagination of the lysosome membrane. This is important to eliminate intracellular pathogens when they enter the cells via phagocytosis. This is degraded upon phagosome fusion with a lysosome to form a phagolysosome (28). However, the degradation pathway may fail, and pathogens can escape the phagosome and enter the cytoplasm. In this condition, the autophagic machinery is activated, and the autophagosome engulfs the pathogen and fuses with the lysosome for degradation (29). Chaperone-dependent autophagy is a highly selective process where proteins are marked and transported to autophagosomes for degradation. The protein chaperones including p62 and HSP70, deliver these proteins to the autolysosome through translocation complexes in the membrane (30). Macroautophagy is the most common type of autophagy. It is a multi-step process controlled by proteins from the ATG family. A double membrane (phagophore) is formed around cytoplasmic material and then through recruitment of ATG family members including LC3 for an autophagosome. The generation of the autophagosome then fuses with lysosomes to form an autolysosome that degrades the cytoplasmic material (31). The cytoplasmic material could be macromolecules such as proteins and lipids but could also contain organelles. One organelle example is damaged mitochondria by being engulfed in autophagosomes and degraded in autolysosomes. This process is termed mitophagy (32). This process is repeated, forming an autophagy flux to maintain cellular homeostasis to ensure cell survival under stressful conditions such as starvation and oxidative stress. If autophagy is prolonged or the number of autolysosomes increases too fast, cells will undergo cell death, termed autophagy-mediated cell death. The balance will determine whether cellular homeostasis is beneficial to the organism. Cancer cells exploit autophagy to degrade and recycle cellular components, including damaged organelles like mitochondria (mitophagy), to maintain energy production and promote survival (33). This adaptation is particularly important in hematological malignancies such as leukemia, where rapidly proliferating cells face metabolic stress.

### 2.3 The function of lysosome in immunity

The final step in phagocytosis requires fusion between the phagosome and the lysosomes. Phagocytes such as neutrophils, release the lysosomal enzyme elastase, which has a proteolytic activity that can degrade bacterial components and endogenous elements such as matrix proteins. Elastase is released in response to inflammatory signals such as cytokine TNF and lipidic mediators such as leukotriene B4. The endosomal receptor TLR9 can also be activated by mitochondrial DNA following mitophagy, leading to SNARE protein recycling and supporting autophagic flux. Antigen-presenting cells (APC), endocytosis and phagocytosis are linked to antigen processing on major histocompatibility class II molecules (MHC-II) (34). Antigens are taken up by phagocytosis or, in the case of B cells, through surface receptor-triggered endocytosis and can be processed on MHC-II molecules and MHC-1 (35). Lysosomal exocytosis is also important in immune cells. Lysosomal exocytosis also occurs at the B cell synapse formed with APCs, such as follicular dendritic cells that present native particulate antigens at their surface (36). Autophagy regulates inflammation by limiting the production of ROS and the activity of inflammasome. Dysregulated lysosomal function in leukemia cells can impair phagocytic activity and antigen processing, weakening immune surveillance and promoting chronic inflammation (37).

### 2.4 Role of lysosomes in hematopoiesis

mTOR (mechanistic target of rapamycin) signaling pathway is critical for maintaining the balance between Hematopoietic Stem and Progenitor Cells (HSPC) quiescence and activation (38). In their quiescent state, HSCs predominantly rely on glycolysis for energy production rather than mitochondrial oxidative phosphorylation (OXPHOS). Lysosomal activity influences this metabolic preference, thereby preserving HSC quiescence. Lysosomes participate in the degradation of ferritin, a process known as ferritinophagy, to release iron (39). Iron is essential for erythropoiesis (red blood cell production). Autophagy modulates the differentiation of HSPCs into various blood cell lineages, such as erythrocytes, granulocytes, and lymphocytes. For example: In erythropoiesis, autophagy helps degrade mitochondria (mitophagy) during the maturation of red blood cells. In lymphopoiesis, autophagy supports T and B cell development by regulating organelle turnover and metabolic reprogramming. During hypoxia or nutrient deprivation, autophagy protects HSPCs by reducing oxidative stress and maintaining cellular homeostasis (40).

Autophagy-related genes, such as ATG5, ATG7, and Fip200, are essential regulators of hematopoiesis. ATG5 and ATG7 are crucial for autophagy initiation and support HSPC quiescence and survival, particularly under stress conditions (41–43).Fip200, involved in autophagosome formation, is key to HSPC differentiation into various blood cell lineages, including erythrocytes, granulocytes, and lymphocytes (44). Disruption of these genes impairs hematopoietic differentiation and increases susceptibility to hematologic diseases, underscoring their vital role in maintaining hematopoietic homeostasis. For instance, mice lacking functional autophagy genes, including FIP200, Atg7, and Atg12, experience a severe loss of hematopoietic stem cells (HSCs), leading to conditions like anemia and leukemia (45). These findings underscore the vital role of autophagy in maintaining hematopoietic homeostasis.

### 2.5 Role of lysosome in the secretory pathway

Cells have a strategic alternative to lysosomal degradation to dispose of the waste through either an autophagic machinery-dependent manner, the process is defined as secretory autophagy, lysosomal-exocytosis-or lysosome derived ectosomes. Autophagy-related secretory pathways called LC3-dependent EV loading and secretion (LDELS) capture proteins at late endosomes. It facilitates their secretion outside the cell (46). Lysosomal exocytosis is the fusion of mature lysosomes to the plasma membrane allowing lysosomal contents to be released into the extracellular space. Lysosomal ectosomes are formed when lysosomes fuse with the plasma membrane forming an outward budding microvesicle. The lysosome ectosomes are then released into the extracellular space. These lysosomes’ secretory pathways release their enzymatic contents into the extracellular space and influence the cellular microenvironment. For example, lysosomal enzymes degrade extracellular matrix components, facilitating cell migration and dissemination. Furthermore, the pro-inflammatory cytokine lacking signal peptide IL1β is delivered to the extracellular space by autophagy-based UPS mechanism and stimulation of autophagy leads to inflammasome-dependent IL1β secretion (47, 48). In leukemia, secretory autophagy and lysosomal exocytosis contribute to the dissemination of malignant cells by releasing enzymes that degrade the extracellular matrix, facilitating tumor invasion and metastasis. Additionally, the secretion of pro-inflammatory cytokines like IL1β through these lysosomal pathways can promote chronic inflammation in the tumor microenvironment, supporting leukemia progression and immune evasion (49, 50).

### 2.6 Lysosomal-mediated cell death

When lysosomes are ruptured, lytic content is released into the cytoplasm such as hydrolases and cathepsins through a mechanism called lysosomal membrane permeabilization (LMP). This triggers cell death often coined lysosome-mediated cell death (51). Lysosomal membrane damage can be induced by free radicals, lysosomotropic agents, endogenous pore-forming proteins, and accumulation of sphingomyelin or protein aggregates (52). LMP induces apoptosis mediated by cathepsins. Cathepsins cleave pro-apoptotic member Bid into an active truncated form (tBid) that translocates to the mitochondria leading to pro-apoptotic Bcl-2 family members BAX and BAK oligomerization (53). This leads to cytochrome c release, activation of caspases and cell death. In addition, cathepsins degrade anti-apoptotic proteins Bcl-2, Bcl-xl and Mcl-1 promoting apoptosis. Besides cathepsins, reactive oxygen species (ROS) are generated following LMP due to the release of acidic contents in lysosomes (54). This leads to mitochondrial membrane permeabilization, caspase activation, and apoptosis. Besides apoptosis, LMP leads to necrosis-like cell death through the proteolytic disintegration of cellular organelles (55). Pyroptosis is an inflammatory regulated form of cell death where the inflammatory protein complex (inflammasome) activates pro-caspase 1 and induces an inflammatory response through the IL1β and IL18 cleavage. LMP induced by ROS or protein aggregates releases cathepsins and induces the inflammasome leading to pyroptosis (56). Lysosomes contain reactive iron that upon LMP induces lipid peroxidation leading to iron-mediated cell death (ferroptosis) (57). Autophagy-induced cell death can be activated when the effector mechanisms of apoptosis are inhibited, either due to the presence of caspase inhibitors or the double knockout of BAX and BAK (58, 59). Excessive engulfment of cytoplasmic material, including mitochondria, through autophagy, leads to cell death. LMP participates through further induction of autophagy flux through ROS, and accumulation of cholesterol and ceramide in lysosomes causing membrane disruption and release of lysosomal contents (60). When lysosome rupture exceeds lysosome biogenesis, it can also inhibit autophagy flux and disrupt autophagy survival function (61). The regulation between the survival and cell death function of autophagy still needs to be elucidated. In the context of leukemia, LMP-induced cell death could be integrated into existing treatment strategies by using agents that promote lysosomal destabilization, thereby sensitizing leukemia cells to chemotherapy or other therapies. These agents, such as lysosomotropic drugs or compounds that induce ROS production, could enhance the cytotoxic effects of standard treatments by amplifying LMP, accelerating tumor cell death, and overcoming treatment resistance (62).

## 3 Dysfunctional lysosomes in hematological malignancies

A cancer cell contains more active lysosomes than a healthy cell and demonstrates high levels of mTORC1 signaling, catabolic reactions and autophagy which aid in cancer cell survival, metabolism and proliferation (63). Lysosomal neuraminidase (NEU1) enhances lysosomal exocytosis and lysosomal hydrolase activity, remodeling the extracellular matrix within the tumor and invading the neighboring tissue thus promoting cancer metastasis (64). Metastatic cells are more vulnerable to lysosome-targeting drugs because lysosomes are highly diverse in size, content, location and activity compared to normal cells (65). However, this lysosomal heterogeneity also poses a challenge in treatment, as chemotherapeutic drugs often become sequestered within lysosomes, contributing to chemoresistance (66).

**Figure 2:**
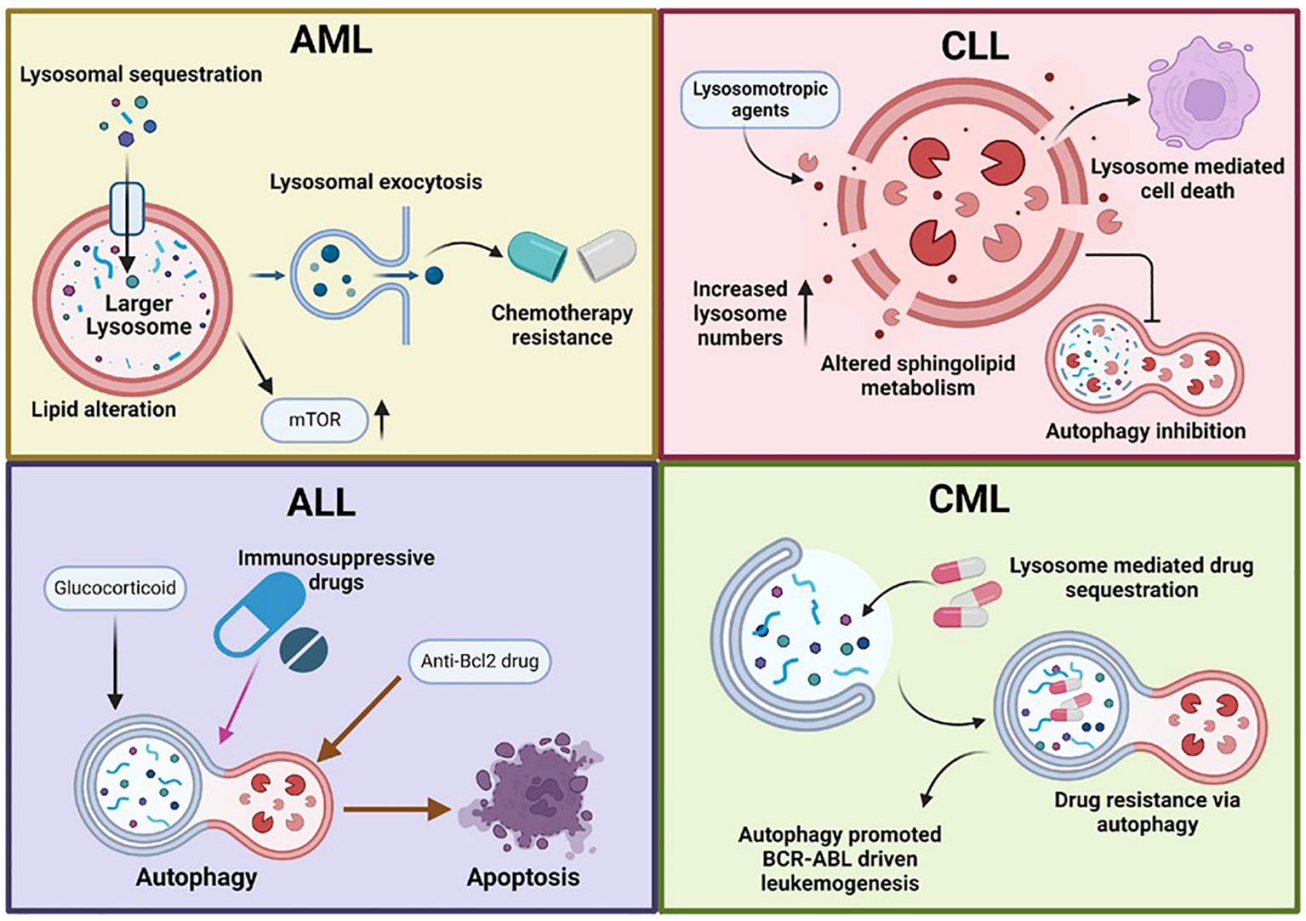
Lysosomal dysfunction in hematological malignancies. This figure illustrates the role of dysfunctional lysosomes in various hematological malignancies, including acute myeloid leukemia (AML), acute lymphocytic leukemia (ALL), chronic myeloid leukemia (CML), and chronic lymphocytic leukemia (CLL). Lysosomal dysfunction in cancer cells is characterized by increased lysosomal biogenesis, larger lysosomes, enhanced lysosomal activity, and altered autophagy pathways. These abnormalities promote cancer cell survival, proliferation, and drug resistance. AML cells often have larger, more active lysosomes that enhance autophagy, promoting survival and proliferation. These include drug sequestration, altered acidification, mTORC1 signalling, and changes in lipid profiles. Lysosomal exocytosis also aids in extracellular remodelling, supporting tumour invasion and metastasis. In ALL, autophagy is reduced in BCR-ABL+ cells. Immunosuppressive and glucocorticoid drugs induce autophagy, while mTORC1 inhibition can trigger autophagy-mediated cell death. Anti-Bcl-2 drugs also cause autophagy-dependent cell death through the ATG5 pathway. In chronic lymphocytic leukemia (CLL), lysosomes play a key role in BCR signaling and cell death. There is altered sphingolipid metabolism, increased lysosome numbers, and selective disruption by lysosomotropic agents. Additionally, autophagy is inhibited in CLL. In CML, autophagy contributes to drug resistance and promotes BCR-ABL-driven leukemogenesis. Additionally, lysosomes mediate drug sequestration, further aiding in resistance mechanisms.

Autophagy plays a critical role in maintaining cellular homeostasis by degrading damaged proteins and organelles. In the context of myeloproliferative neoplasms, specific oncogenes such as JAK2^V617F^, a mutation commonly found in polycythemia vera, essential thrombocythemia, and myelofibrosis, may interact with autophagic pathways (67). In addition to JAK2 (Janus kinase 2), MPL plays a significant role in the pathogenesis of myeloproliferative neoplasms (MPN), particularly in essential thrombocythemia (ET) and myelofibrosis (MF) (68). MPL is a crucial oncogene signalling protein that functions as a receptor for thrombopoietin (TPO), a key regulator of platelet production (69). Mutations in MPL, particularly MPLW515L/K, have been identified in a subset of MPN cases, contributing to the dysregulation of hematopoiesis and abnormal megakaryocyte proliferation (70). These mutations lead to constitutive activation of MPL signalling pathways, promoting excessive megakaryocyte production and platelet overproduction, hallmarks of ET and MF. MPL also undergoes autophagic degradation, particularly in the context of JAK2 and CALR mutations, which are common in MPN (71). In these mutated forms, MPL interacts with the autophagic machinery, contributing to the regulation of protein homeostasis within the cell. Autophagic degradation of MPL serves as a mechanism to modulate its signalling activity, preventing excessive signalling that could lead to uncontrolled cell proliferation. Dysregulation of this autophagic process may contribute to the pathogenesis of MPN by allowing for sustained MPL signalling, leading to the expansion of abnormal hematopoietic progenitors (72). Thus, the interplay between MPL, JAK2 and autophagy highlights the complex role of autophagic pathways in regulating oncogene signaling in MPNs. Mutations or balance, dysfunction that disrupt this balance may promote hematopoietic malignancy. Understanding the role of autophagic degradation involving lysosomes in these neoplasms could provide new insights into potential treatment strategies (**Figure 2**).

Most studies have focused on solid tumors, but lysosomes contribute to the development of various hallmarks of hematological malignancies, such as sustained proliferative signaling (mTORC1 signaling), metabolism (catabolic reactions, autophagy) and invasion (lysosomal exocytosis) (73). Furthermore, lysosome-targeted therapies have shown promise in hematological malignancies, particularly leukemia, where they induce cytotoxic effects on leukemia cells (**Table 1**).

**Table 1:** Lysosomotropic agents and their mechanisms of action in hematological malignancies.

Agent	Type	Mechanism of Action	Target Malignancy	References
**Mefloquine**	Lysosomotropic Agent	Elevates lysosomal pH, leading to lysosomal dysfunction and cell death.	AML	(74)
**Cationic Amphiphilic Drugs (CADs)**	Lysosomotropic Agents	Accumulate in lysosomes, altering lipid profile and inducing lysosomal cell death.	AML	(75)
**Quercetin**	Flavonoid Compound	Induces lysosomal cell death through disruption of lysosomal function.	AML	(76)
**Dp44mT**	Metal Chelator	Accumulates in lysosomes, induces lysosomal cell death, and triggers mitochondrial apoptosis.	AML	(77, 78)
**Archazolid A**	V-ATPase Inhibitor	Inhibits lysosomal acidification, disrupting lysosomal function.	AML	(79)
**Deoxysappanone B 7, 4′-Dimethyl Ether (Deox B 7, 4)**	Microtubule Inhibitor	Enhances lysosomal V-ATPase activity, inducing lysosomal hyper-acidification and apoptosis.	AML	(80)
**Siramesine**	Lysosomotropic Agent	Inhibits acidic sphingomyelinase, disrupting lysosomal function and inducing cell death.	CLL	(62, 81)
**Hydroxychloroquine**	Autophagy Inhibitor	Inhibits lysosomal acidification, disrupting autophagy and inducing cell death.	CLL	(82)
**Chloroquine**	Autophagy Inhibitor	Prevents lysosomal acidification, leading to autophagosome accumulation and cell death.	CLL	(83)
**Lys05**	Autophagy Inhibitor	Accumulates within lysosomes, causing significant lysosomal dysfunction and cell death.	CML	(84)
**Bafilomycin A1**	V-ATPase Inhibitor	Prevents lysosomal acidification, inhibiting autophagic flux and inducing apoptosis.	CLL	(85)
**Niclosamide**	Anti-helminthic Drug	Disrupts lysosomal function, inducing cell death through lysosomal destabilization.	CLL	(86)
**Obatoclax**	Bcl-2 Inhibitor	Induces autophagy-dependent cell death, specifically in an ATG5-dependent manner.	T-ALL	(87)
**FTY720**	Immunosuppressive Drug	Induces autophagy and contributes to cell death in B-ALL cells.	B-ALL	(88)

This table summarizes various lysosomotropic agents used in the treatment of hematological malignancies. Each agent is categorized by type, with a description of its mechanism of action, targeted malignancy, and supporting references. These agents primarily function by disrupting lysosomal integrity or inhibiting autophagy, leading to cancer cell death across different hematologic malignancies such as AML, CLL, CML, T-ALL, and B-ALL.

### 3.1 Acute myeloid leukemia

Acute Myeloid Leukemia (AML) is a hematological malignancy characterized by an excessive accumulation of immature blood-forming cells in the bone marrow, impairing the formation of normal blood cells (89). Treatment is tailored to cytogenetic risk, consisting of induction chemotherapy with cytarabine and anthracycline plus gemtuzumab-ozogamicin (IgG4-kappa monoclonal antibody linked to a cytotoxic drug) or FLT3-inhibitor midostaurin in young fit adult patients and HDAC inhibitor azacitidine with or without BCL2-inhibitor venetoclax in older or unfit adults (90). Consolidation therapy occurs after initial remission and usually involves additional rounds of treatment. Finally, allogeneic stem cell transplantations are performed if needed for certain subtypes when there is a suitable donor. A subtype of AML is acute promyelocytic leukemia (APL) and is treated with all-trans retinoic acid (ATRA) and either arsenic trioxide or anthracyclines (91).

Using gene clusters, it was demonstrated that AML cells with higher lysosome-related genes predict the prognosis of AML cells and response to chemotherapy. AML cells also show larger lysosomes and increased expression of lysosome biogenesis genes indicating higher lysosome activity (92). Indeed, increased lysosome activity supports the survival of senescent AML cells. Furthermore, the expression of transient receptor potential mucolipin (TRPML1) found in lysosomes is upregulated in AML cells (93). In contrast, lysosome biogenesis transcription factor TFEB acts as a tumor suppressor that induces differentiation and cell death in normal and malignant myeloid progenitor cells, thus controlling myelopoiesis (94). This has been linked to its role in MYC signaling and epigenetic controls and not to its function in lysosomal biosynthesis. This indicates that AML cells have altered lysosomes to elevated biomass and bioenergetic demands.

Besides altered lysosomal biosynthesis, autophagy plays an important role in AML progression. Several common chromosomal deletions in AML include regions of autophagy genes. Depletion of autophagy gene ATG5 led to increased proliferation and more aggressive leukemia in the MLL-ENL-induced murine AML model. Deletion of ATG7 in OCI-AML3 cells also increased chemotherapy resistance in xenograft mice (95). In addition, inhibition of autophagy, followed by deletion of ATG5 or ATG7, reduces leukemia-initiating cells (LICs) and increases mitochondrial ROS (96). Inhibition of autophagy also promotes the survival and proliferation of AML cells (97). Mutations in SQSTM1/p62 are believed to impact mitophagy and myeloid leukemia development (98). In FLT3-ITD-driven AML, mTORC1 activation suppresses autophagy and prevents the autolysosome degradation of the FLT3 protein. In contrast, FLT3-ITD upregulates transcription factor ATF4 increasing basal autophagy in AML cells and inhibiting autophagy increasing survival of the FLT3-ITD-driven AML mice (99). This illustrates the multiple role autophagy plays in AML progression.

Targeting lysosomes is one of many strategies to combat chemoresistance. Lysosomotropic agents are chemicals that accumulate in the lysosomal lumen and elevate lysosomal pH, leading to lysosomal dysfunction and membrane permeabilization (LMP). Mefloquine, a lysosome-damaging agent, releases lysosomal CTSB and CTSL into the cytosol, inducing cell death in AML cells (74). Similarly, cationic-amphiphilic antihistamines target leukemia cells in patients (100). Archazolid A, a V-ATPase inhibitor, has shown anti-leukemic effects by suppressing lysosomal acidification (79, 80). Conversely, the microtubule inhibitor deoxysappanone B 7, 4′-dimethyl ether (Deox B 7, 4) demonstrates anti-leukemic activity by enhancing lysosomal V-ATPase activity, resulting in hyper-acidification of lysosomes and inducing apoptosis in AML cells (80). Cationic amphiphilic drugs (CAD) are another category of small molecules that accumulate in lysosomes, altering the lipid profile in the lysosomal lumen and inducing lysosomal cell death in multiple AML cell lines (75). The flavonoid quercetin, a polyphenol compound, induces lysosomal cell death in leukemia cells (76). The metal chelator Dp44mT, which accumulates in lysosomes, induces lysosomal cell death (77). Dp44mT also triggers the release of CTSD from lysosomes into the cytosol, initiating mitochondrial cytochrome-c-dependent apoptosis (78, 101). In summary, lysosomal acidification plays a significant role in AML and targeted therapeutic approaches can be selected based on the status of lysosomal acidification (102).

### 3.2 Acute lymphocytic leukemia

Acute lymphocytic leukemia (ALL) belongs to a highly heterogeneous group of hematological malignancies from the lymphoid lineage. ALL is characterized by uncontrolled proliferation of clonal neoplastic cells in the bone marrow leading to bone marrow failure and death if untreated. B cell-derived ALL (B-ALL) represents 80% of all ALL cases (103). It affects mostly children but can occur in adults. T cell-derived ALL (T-ALL) accounts for 10-15% of all ALL cases and is characterized by uncontrolled proliferation of lymphoblast arising from the thymus (104). Intensive combination chemotherapy has improved survival, particularly in children, but drug resistance still occurs (105). Newer agents include bispecific T cell engager (CD19-CD3) blinatumomab and antibody (anti-CD22) - drug conjugate inotuzumab. Allogeneic stem cell transplantation or CAR-T cell therapy is used in high-risk and refractory cases.

The role of lysosomes in ALL has focused on autophagy in the survival and death of leukemia cells. In B-ALL, cells with the p185 form of BCR-ABL show reduced basal levels of autophagy, but when autophagy was blocked by the knockdown of ATG3, cells underwent apoptosis. The immunosuppressive drug FTY720 induced cell death in B-ALL cells in a caspase-independent manner but also induced autophagy, contributing to cell survival (88). In contrast, Glucocorticoids (GC) have been shown to induce cell death through the activation of the autophagic machinery in B-ALL cell lines and primary cells (106, 107). Using a mTORC1 inhibitor everolimus, autophagy was increased in B-ALL cell lines and primary samples. Knocking down Beclin-1 resulted in less everolimus cytotoxicity suggesting autophagy provided a cell death response. Nevertheless, using an autophagy inhibitor, 3-methyladenine failed to block everolimus-induced cell death in B-ALL cells. Obatoclax induces autophagy-dependent cell death in ALL, specifically in an ATG5-dependent but Beclin-1-independent manner (87). Taken together, the context of autophagy activation in B-ALL cells will determine whether autophagy contributes to cell survival or death.

In T-ALL cells, inhibition of either PI3K/mTOR or Akt pathways has been shown to induce autophagy. Treatment of T-ALL cells with AKT inhibitors demonstrated that autophagy was protective as shown by knockdown of Beclin or using autophagy inhibitor chloroquine. Autophagy may also play an active role in the cell death of T-ALL cells, as evidenced by a study where Jurkat cells were treated with selenite, a drug known for its anti-tumor efficacy, exerting both pro-apoptotic and pro-autophagic effects (102). Metformin (LKB/AMPK inhibitor) induced autophagy contributed to cell death in T-ALL cells. Using the anti-Bcl-2 drug obatoclax in GC-resistant T-ALL cell lines showed increased autophagy mediated by dissociation of Beclin-1 from Bcl-2 family member Mcl-1 and reduced mTORC1 activity (108). This led to an autophagy-dependent necroptosis in these cells. Similar to B-ALL, the context of autophagy activation will determine whether it contributes to cell survival or death.

### 3.3 Chronic myeloid leukemia

Chronic myeloid leukemia (CML) is a myeloid-derived leukemia characterized by the expression of the BCR: ABL fusion oncoprotein. This constitutively active tyrosine kinase leads to uncontrolled proliferation and growth. Using ABL tyrosine kinase inhibitors (TKI) such as imatinib and newer generation TKIs has proven to be an effective therapy however drug resistance remains a problem. The lysosome sequestration of hydrophobic weak base drugs such as imatinib could be the mechanism of drug resistance but no imatinib-resistant CML cell line has shown lysosomal mediated resistance (109). Autophagy is another lysosome-regulated mechanism that could contribute to drug resistance. Autophagy is induced in both CML cell lines and Leukemic Stem Cells (LSCs) following *in vitro* treatment with imatinib, suggesting that BCR-ABL acts as a negative regulator of autophagy (110). However, other studies indicate that the expression of this oncogene promotes autophagosome formation and that autophagy is essential for BCR-ABL-dependent leukemogenesis. Combining mTOR and autophagy inhibition has proven effective in targeting TKI-resistant CML cells. In a CML Patient-Derived Xenograft (PDX) model, treatment with talazoparib in combination with autophagy inhibitor chloroquine significantly enhanced the anti-tumour effect of talazoparib (111). Talazoparib markedly triggered autophagy in CML cells, as confirmed by the accumulation of autophagosomes, decreased SQSTM1 levels, and upregulation of LC3-II (111, 112). This suggests autophagy inhibition might be an effective treatment to overcome TKI drug resistance in CML.

### 3.4 Chronic lymphocytic leukemia

Chronic lymphocytic leukemia (CLL) is the most common type of leukemia in the Western world. It is characterized by the accumulation of CD5+ and CD19+ monoclonal B cells. Standard therapies currently utilize a second-generation BTK tyrosine kinase inhibitor such as acalabrutinib or an anti-Bcl-2 mimetic drug, venetoclax alone or in combination with anti-CD20 antibodies. Unfortunately, drug resistance remains a problem. Lysosome-mediated cell death presents a compelling therapeutic avenue for CLL. In CLL cells, lysosomes are involved in regulating the BCR signaling pathway by targeting the TOSO: IgM complex, which is critical for cell survival and proliferation (113). Lysosome numbers are also increased in CLL cells compared to normal B cells. In addition, CLL cells have altered sphingolipid metabolism, leading to increased levels of sphingosine. Sphingosine leads to lysosomal membrane instability. CLL cells also have higher basal levels of autophagy, contributing to cell survival.

Lysosomes are involved in the effectiveness of standard treatment for CLL. We have shown that Venetoclax can act as an autophagy inhibitor by suppressing the expression of ATG12 and in combination with starvation or ibrutinib treatment increased cell death. Rituximab, a type I anti-CD20 monoclonal antibody, targets lysosomes for FcγRIIB- promoted internalization of CD20: anti-CD20 complexes, reducing CD20 expression on the cell surface and contributing to rituximab resistance (114). Conversely, GA101, a type II anti-CD20 monoclonal antibody induces lysosome-dependent cell death through lysosome disruption (115). This indicates that lysosomes play a role in CLL treatments.

Due to the alteration in lysosomes, several lysosomotropic agents have been used to treat CLL cells. Siramesine is a lysosomotropic agent shown to induce cell death in several different types of cancer including breast and brain cancers through its inhibition of the acidic sphingomyelinase (ASMase). In CLL cells, siramesine was shown to selectively target lysosome disruption compared to normal B cells and was effective in combination with venetoclax. Besides siramesine, other lysosomotropic agents such as antihistamines and antimalarial drugs induce lysosome-mediated cell death in CLL cells. Furthermore, these drugs synergize with ibrutinib to increase apoptosis. This indicates that lysosomotropic agents could be effective treatments for CLL.

Several drugs targeting the autophagy pathway through inhibiting autolysosomes induce cell death in CLL cells. Hydroxychloroquine, traditionally used for malaria and autoimmune diseases, inhibits lysosomal acidification, disrupting autophagy and promoting CLL cells (116). Similarly, chloroquine prevents lysosomal acidification, leading to the accumulation of autophagosomes and cell death in CLL cells (83). Lys05, a more potent derivative of chloroquine, accumulates within lysosomes, causing significant lysosomal dysfunction and resulting in the death of CLL cells (84). Bafilomycin A1, a V-ATPase inhibitor, prevents lysosomal acidification, leading to the inhibition of autophagic flux and apoptosis in CLL cells (85). Niclosamide, an anti-helminthic drug, disrupts lysosomal function and autophagy, inducing cell death in leukemia cells through mechanisms involving lysosomal destabilization (86). These studies suggest that targeting lysosome function in autophagy might render CLL cells sensitive to cell death.

## 4 Future perspectives

Dysfunctional lysosomes play a critical role in the pathogenesis and treatment resistance of various leukemias, including AML, ALL, CML, and CLL (**Figure 2**). Understanding these mechanisms offers valuable insights into developing targeted therapies that can enhance treatment efficacy and improve patient outcomes in these hematological malignancies. Ongoing research into lysosomal functions and their regulation holds promise for identifying novel therapeutic strategies to combat leukemia and lymphoma (**Table 1**).

Several lysosomal drugs are currently in clinical trials for the treatment of leukemia, targeting lysosomal pathways to induce apoptosis or enhance the efficacy of existing therapies. Venetoclax, a BCL-2 inhibitor, disrupts mitochondrial and lysosomal integrity and is approved for chronic lymphocytic leukemia (CLL) and acute myeloid leukemia (AML), with ongoing trials exploring its use in combination therapies. Hydroxychloroquine, a lysosomal autophagy inhibitor, is in Phase 1/2 trials for various leukemia types, often combined with Venetoclax or chemotherapy to overcome drug resistance (117). Similarly, chloroquine, another autophagy inhibitor, is in early-phase trials and shows potential to enhance the efficacy of standard therapies in AML and CLL (118). Dactinomycin, known for inducing lysosomal stress, is approved for acute lymphoblastic leukemia (ALL) and remains a part of combination regimens for pediatric cases (119). Lysosome-targeted therapies are emerging as a promising approach in the treatment of leukemia, particularly due to the critical roles lysosomes play in cell survival, apoptosis, and immune evasion. Research is ongoing to identify optimal combinations and treatment schedules to maximize the benefits of lysosome-targeted therapies. Biomarker-driven approaches may help tailor these therapies to individual patients, improving efficacy and minimizing adverse effects. By understanding the roles of lysosomal dysfunction in leukemia pathogenesis, therapeutic strategies can potentially enhance treatment outcomes, overcome resistance mechanisms, and provide a more effective means of targeting this heterogeneous group of malignancies. Clinical trials of lysosome-targeted agents alone and in combination with standard therapies will be crucial for advancing these innovative approaches and improving patient care in leukemia.

## 5 Conclusion

Lysosomes play important functions within cells beyond just maintaining cellular homeostasis. In hematological malignancies, lysosomes are altered to maintain increased demand for biomass and bioenergetics leading to cell survival and growth. Disrupting the lysosomal membrane integrity or modulating lysosomal enzyme activity has shown a potential to selectively induce cell death in leukemia cells. The efficacy of lysosome-mediated cell death in pre-clinical studies suggests that it could be a valuable addition to the existing therapies, to overcoming resistance and improving patient outcomes. Further clinical trials are necessary to fully understand the safety and effectiveness of these interventions in the context of hematological malignancies.
